# A modified percutaneous atrial balloon septoplasty for difficult transseptal puncture

**DOI:** 10.1097/MD.0000000000026525

**Published:** 2021-07-16

**Authors:** Siyu Wang, Lei Zhao, Yuxing Wang, Xiandong Yin, Xinchun Yang, Ye Liu

**Affiliations:** Heart Center & Beijing Key Laboratory of Hypertension, Beijing Chaoyang Hospital, Capital Medical University, Beijing, China.

**Keywords:** atrial fibrillation, balloon, catheter ablation, septoplasty, transseptal

## Abstract

Catheter ablation of atrial fibrillation sometimes encounters difficulty in passing the interatrial septum. This study reports a modified percutaneous atrial balloon septoplasty with short balloon to gain access to left atrium (LA) during challenging transseptal puncture (TSP).

We retrospectively analyzed 20 patients (61.75 ± 7.31 years, 45% male) who received modified percutaneous atrial balloon septoplasty from August 2015 to October 2018. Soft-headed balance middle weight (BMW) guidewire was inserted into left superior pulmonary vein (LSPV) and short non-compliant balloon (15 mm in length and 4.0 or 5.0 mm in diameter) was used for atrial balloon septoplasty (ABS). Interatrial septum was located with inflated balloon and contrast “Hitting Wall” sign. All patients were followed-up for iatrogenic atrial septal defect (iASD) and other related complications.

ABS and LA access were performed successfully without complications in all 20 patients. Time needed for ABS was correlated to the number of prior TSP (*P* = .007). During the 6-month follow-up, no remaining iASD was found by echocardiography.

For atrial fibrillation patients with difficulty in passing the interatrial septum, this modified percutaneous ABS might be an alternative strategy which is safe to obtain transseptal access without short or long term complications.

## 1 Introduction

Catheter ablation procedure is the most common treatment for atrial fibrillation (AF) or other left atrial (LA) interventions and its number has increased dramatically in recent years. Pulmonary vein isolation (PVI) is the cornerstone of AF ablation and transseptal puncture (TSP) is a critical step in achieving LA access.^[1]^ However, site of TSP in some patients can be abnormally thick or fibrotic, particularly in those with scarred, surgical, or percutaneous repaired atrial septum. Previous research reported several methods of tackling difficult TSP which included stiff guidewire perforation, radiofrequency powered flexible needle and deep inspiration maneuver.^[2–4]^ The most widely accepted method is atrial balloon septoplasty or septostomy (ABS), but the documented procedures of ABS varies and it may pose potential adverse events.^[5,6]^ In this study we performed coronary angioplasty while introducing a modified safer way of ABS. We reported a series of patients in whom percutaneous ABS was performed for successful TSP during catheter ablation of AF.

## 2 Materials and methods

### 2.1 Study population

The institutional review board of Beijing Chaoyang Hospital approved this retrospective observational study. All patients received informed consent, and the study protocol conforms to the ethical guidelines of the 1957 Declaration of Helsinki as reflected in a prior approval by the Institution's Human Research Committee. A total of 1230 patients underwent AF ablation in our hospital between August 2015 and October 2018 were included. Among these patients, 20 received modified percutaneous ABS because of the presence of difficulty in passing the interatrial septum. Demographic information and clinical features of all patients were collected from electronic medical records (EMR). All of the participants met our criteria and were hospitalized with or without a diagnosis of atrial fibrillation. Paroxysmal AF was determined as AF episodes that were self-terminating or converted within 7 days. Persistent AF was classified as AF that lasted longer than 7 days, including episodes that were terminated by cardioversion after 7 days or more. The patients received novel oral anticoagulants before the procedure and trans esophageal echocardiography (TEE) was used to exclude thrombosis in left atrial appendage. 9 patients were taking statins due to pre-existing coronary artery disease. Two paroxysmal atrial fibrillation patients had a history of amiodarone use.

### 2.2 Ablation procedure

Left atrial mapping and ablation procedures have been described in previous studies.^[1]^ Preoperative TEE was completed to exclude left atrial appendage thrombus and the presence of patent foramen ovale (PFO) or ASD. Patients were transported to catheterization room in fasting state. Local anesthesia was achieved with lidocaine, and femoral access of both legs were established with Seldinger technique. Deflectable decapolar catheters were positioned in the coronary sinus via left femoral vein. Transseptal puncture and atrial balloon septoplasty were described later. Electroanatomic mapping of left atrium was performed using Pentaray high-density Mapping Catheter (Biosense Webster). Thermocool SmartTouch SF Catheters were used for radiofrequency (RF) ablation. Antral PVI was achieved in all procedures, and linear ablation was added if necessary. In 2 patients, cryoballoon ablation with 28 mm Arctic Front Advance (Medtronic) was used for PVI. Detailed procedure was described in other studies.^[2]^ Cryoablation catheter was delivered to the left atrium. The balloon catheter was inflated at the antrum of each vein and advanced until contact with the pulmonary vein ostium was made. Two 180 s freezes were targeted for each pulmonary vein.

### 2.3 Transseptal puncture

**Figure 1:**
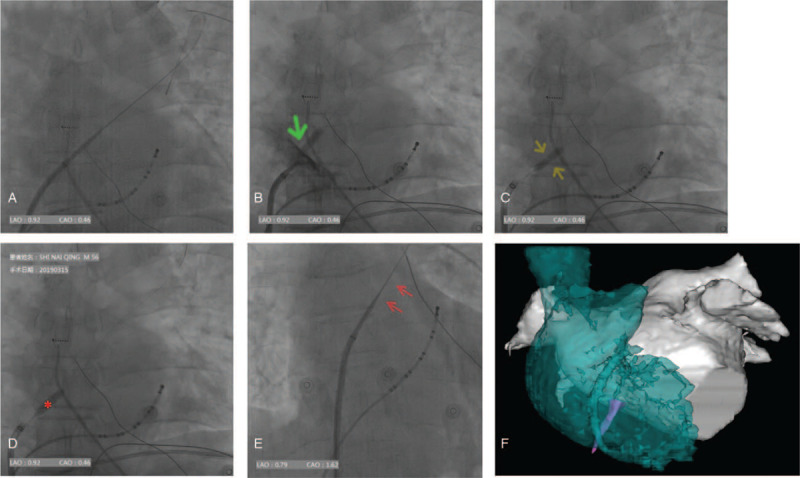
Process of modified ABS. Swartz sheath failed to pass interatrial septum with routine TSP procedure. Coronary angioplasty balloon inflated and contrast injected. The green arrow indicates the “Hitting Wall” sign--the position of interatrial septum. The guidewire has been switched to soft head BMW and dilator of the sheath was removed. Balloon atrial septoplasty. The “waist” of inflated balloon (yellow arrows) represented the interatrial septum. The balloon was reinflated several times and the “waist” disappeared (red asterisk) indicating a successful ABS. TSP sheath assembly passes through interatrial septum after septoplasty via 0.032” J-tip guidewire (red arrows). Merged images from cardiac CT reconstruction and CARTO FAM. The magenta structure represents the position of angioplasty balloon and the route of TSP. ABS = atrial balloon septoplasty, TSP = transseptal puncture, FAM = fast anatomic mapping.

TSP was performed from the right femoral vein. For RF cases, an 8.5Fr 63 cm Fast-Cath transseptal guiding introducer Swartz sheath SL1 (St. Ju vde Medical) was advanced over a 0.032” 180 cm J-tip guidewire to the superior vena cava under fluoroscopic guidance. Flushed Brockenbrough transseptal needle (BRK-1 XS, St Jude Medical Spec Sheet) was introduced into SL-1 sheath. The assembly was withdrawn to optimal TSP site under fluoroscopy. Interatrial septum was successfully punctured with brockenbrough needle then advancement of the transseptal dilator over the needle was possible in all cases. J-tip guidewire was then placed into the left superior pulmonary vein to support SL1 catheter to enter left atrium. When Swartz sheath failed to pass interatrial septum with routine TSP procedure, the procedure of modified balloon septoplasty in this study was performed (Fig. 1A).

### 2.4 Atrial balloon septoplasty

With Brockenbrough needle tip inserted into LA, TSP assembly was advanced into LA to cover the needle tip. A softheaded 0.014“×190 cm coronary guidewire Balance middle weight (BMW) Universal II (Abbott Vascular) was positioned in the left superior PV via needle pore. The needle and dilator were carefully withdrawn leaving the sheath in the right atrium and BMW guidewire in the LSPV. A non-compliant rapid exchange NC Sprinter angioplasty balloon with diameter of 4.0 mm (5.0 mm for cryoballoon ablation) and length of 15 mm (Medtronic, Inc) was advanced through BMW guidewire into LA and inflated with contrast saline. The inflated balloon was then withdrawn until blocked by interatrial septum, and contrast was injected through the sheath. Atrial septum was confirmed when contrast displayed a “Hitting Wall” sign and dissipated (Fig. 1 B). The balloon was deflated and repositioned in the section of septum and was inflated with nominal pressure 10 atm (1000kPa) for 10 seconds. A “waist” of about 70% of the width of balloon was visualized over the septum under fluoroscopy (Fig. 1 C). The balloon was reinflated several times until the “waist” disappeared (Fig. 1 D) to dilate the fibrotic septum and create enough room for transseptal sheath assembly to slide into LA. The balloon was then deflated and withdrawn. 0.032” J-tip guidewire was exchanged for BMW angioplasty guidewire and was advanced into LSPV. Sheath dilator was reintroduced over it. The SL-1 sheath assembly was then passed into middle cavity of LA (Fig. 1 E). For cryoballoon cases, a FlexCath Advance steerable sheath (12-F inner diameter, 15-F outer diameter; Medtronic) was advanced over 0.032” 180 cm J-tip wire positioned in the left superior PV after LA access was initially obtained with an SL-1 sheath. The procedural workflow of atrial balloon septoplasty was summarized in Figure 3.

### 2.5 Follow-up

After ablation, patients were carefully monitored in wards for perioperative complications for at least 2 days. Routine follow-up was arranged at the first and 6th month after the operation. Transthoracic echocardiography was performed at the 6th month on every recurrent patient for identifying obvious iatrogenic atrial septal defects (iASD).

### 2.6 Statistical analysis

Descriptive analysis was expressed as mean ± standard deviation for continuous variables and percentage for categorical variables. Correlation analysis was performed between each variable. Besides, partial correlation coefficients were used to describe linear relationship between 2 variables while controlling for the effects of other variables. A *P* < .05 was considered statistically significant. R 3.4.2 was used for statistical analysis.

## 3 Results

### 3.1 Patient characteristics

This study included 20 patients received modified percutaneous ABS due to difficult TSP (61.75 ± 7.31 years, 45% male). Mean LA diameter was 44.10 ± 6.32 mm and mean left ventricular ejection fraction was 64.95 ± 6.50%. Demographic characteristics of these patients are listed in Table 1. 6 (30%) patients received TSP for the first time.

**Table 1:** Clinical and demographic characteristics of patients undergoing ABS during AF ablation.

Patient #	Age (yrs)	Sex	HTN	DM	CAD	AF Type	LA Diameter (mm)	EF	Number of Prior TSP	Procedure
1	72	Female	N	N	N	Persistent	42	62	3	Radio Frequency
2	69	Male	Y	N	Y	Persistent	49	68	2	Radio Frequency
3	65	Male	N	Y	N	Persistent	43	70	2	Radio Frequency
4	59	Female	N	N	Y	Persistent	41	51	2	Radio Frequency
5	57	Male	Y	N	N	Persistent	49	67	2	Radio Frequency
6	55	Female	N	N	Y	Paroxysmal	42	65	0	Cryoballoon
7	51	Male	Y	N	Y	Paroxysmal	35	65	0	Cryoballoon
8	58	Male	Y	N	N	Persistent	46	65	2	Radio Frequency
9	53	Female	Y	Y	Y	Paroxysmal	37	57	2	Radio Frequency
10	61	Male	Y	N	Y	Paroxysmal	46	62	1	Radio Frequency
11	61	Female	Y	N	N	Paroxysmal	46	69	0	Radio Frequency
12	75	Male	N	N	N	Paroxysmal	36	66	0	Radio Frequency
13	73	Female	Y	Y	Y	Paroxysmal	36	70	1	Radio Frequency
14	70	Male	Y	N	N	Persistent	50	69	1	Radio Frequency
15	68	Female	Y	Y	N	Persistent	49	68	1	Radio Frequency
16	56	Female	Y	N	N	Persistent	41	66	2	Radio Frequency
17	52	Female	Y	N	Y	Persistent	62	72	0	Radio Frequency
18	60	Male	Y	N	Y	Persistent	48	48	2	Radio Frequency
19	63	Female	N	N	N	Persistent	42	74	1	Radio Frequency
20	57	Female	Y	N	N	Persistent	41	65	0	Radio Frequency

ABS = atrial balloon septoplasty, CAD = coronary artery disease, DM = diabetes mellitus, EF = ejection fraction, HTN = hypertension, LA = left atrium, TSP = transseptal puncture.

### 3.2 Procedure of atrial balloon septoplasty

With J tipped guidewire placed in the left superior PV, repeated attempts to pass into LA with the sheath assembly was unsuccessful due to resistant forces at the interatrial septum. ABS was performed under these circumstances.

Two (10%) patients underwent cryoballoon ablation with larger 15F FlexCath Advance steerable sheath while others received radiofrequency ablation with 8.5F SL1 sheath. ABS was performed immediately after routine TSP technique failed. ABS procedure lasted 14.55 ± 3.71 minutes. The mean total fluoroscopy time was 24.50 (21.00–31.50) minutes. Number of prior TSP experiences was associated with longer ABS time (R2 = 0.58, *P* = .007). Results were shown in Table 2. The correlation between ABS time and prior TSP remained significant after adjusted for AF type (R2 = 0.71, *P* = .001) using partial correlation.

**Table 2:** Correlations of factors for patients receiving modified ABS.

		Correlation			
Variables	Descriptives	ABS time	*P* values	Recurrence	*P* values
Age	61.75 ± 7.31	−0.15	.522	0.14	.555
Male	9 (45%)	0.20	.407	0.05	.833
HTN	14 (70%)	−0.14	.550	0.055	.819
DM	4 (20)	0.06	.794	−0.25	.288
CAD	9 (45%)	0.36	.116	0.105	.660
Persistent AF	13 (65%)	−0.06	.794	0.105	.660
LA Diameter	44.10 ± 6.32	−0.29	.214	0.48	.031
EF	64.95 ± 6.50%	−0.42	.068	0.10	.667
TSP Experiences	2.20 ± 0.95	0.58	.007	0.03	.910
Follow-up Days	115 (97.5, 127.5)	−0.03	.894	0.24	.300

ABS = atrial balloon septoplasty, CAD = coronary artery disease, DM = diabetes mellitus, EF = ejection fraction, HTN = hypertension, LA = left atrium, TSP = transseptal puncture.

### 3.3 Safety and correlation analysis

**Figure 2:**
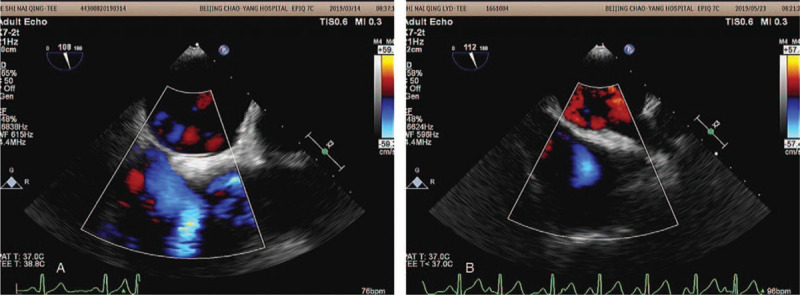
Interatrial septum on TEE of a patient with recurrent AF. TEE result at the first admission before ABS. TEE result at 6-month follow-up with no shunt between 2 atrias. TEE = trans-esophageal echocardiography, AF = atrial fibrillation, ABS = atrial balloon septoplasty.

No procedural complications occurred during hospitalization. After a median follow-up time of 115 days (interquartile range: 97.5 to 127.5 days), AF recurred in 4 (20%) patients including 1 paroxysmal and 3 persistent. Recurred patients tended to have larger LA diameters (R2 = 0.48, *P* = .031). AF recurrence was still significantly related to LA diameter adjusted by AF type (R2 = 0.50, *P* = .028). Transthoracic echocardiography was routinely performed at 6-month follow-up and no remaining iASD was found. 2 of the recurred patients received repeated catheter ablation and ASD was not found in preoperative TEE. Figure 2 showed TEE before ABS and during follow-up of the same patient with recurrent AF.

**Figure 3:**
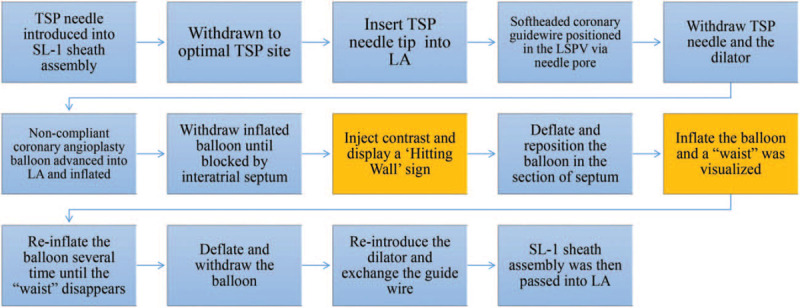
A summarized procedural details of atrial balloon septoplasty. Yellow blocks indicate the 2 steps of localizing the septum. TSP = transseptal puncture, LSPV = left superior pulmonary vein, LA = left atrium.

## 4 Discussion and conclusions

Compared with previous depiction of ABS with stiff-headed guidewire and long balloon or RF needle, this study use soft-headed angioplasty guidewire and short balloon to perform atrial septoplasty under the situation of difficult transeptal puncture. All ABS procedures were successful and transseptal sheath were assembled to enter the LA. We found no procedure related complications which indicated this modified percutaneous ABS is safe and effective and may be an optimal approach in patients with difficult TSP.

There has a growing number of catheter ablation procedures these years because of the increasing incidence and prevalence of AF. With the approximate recurrent rate 30%, thousands of patients need ablation and repeated TSP for multiple times.^[9]^ Difficult TSP occurred in 5% of patients with multiple prior LA ablation procedures and 26% patients with prior atrial septal repair.^[3]^ The puncture site for TSP is called true interatrial septum which is a relatively small area that could be resected without leaving the cavities of the heart.^[4]^ Only the floor of fossa ovalis and its immediate muscular inferoanterior rim build the septum between the right and left atrium.^[5]^ In our study, number of TSP experiences was associated with longer ABS time. As reported by prior research, patients who have undergone prior AF ablation may have septal scarring and thickening at the sites of prior transseptal procedures which made it difficult to access to the LA.^[6]^ Conventional TSP techniques may not be able to cannulate LA. Simply adding brute forces are not able to promote TSP assembling but will increase the chance of perforation leading to cardiac tamponade.^[6]^

Balloon atrial septoplasty is a widely accepted way of obtaining LA access. In one case report, stiff-head guide wire was used to pass into LA and used a long (5 mm × 40 mm) angioplastic balloon for septoplasy.^[6]^ Because of possible impairment of LA with stiff-head guide wire and unavailability of long balloon, this study chose a much safer soft-headed guide wire and a widely used balloon (4.0–5.0 mm × 15 mm). Study from Jackson J incorporated just 15 selected patients and dilation of septum was achieved by using long angioplasty balloons or dilation catheters (diameter 4 to 10 mm, length 2 to 15 cm).^[7]^ Iatrogenic atrial septal defects (iASD) is always the major concern. Selected balloon tends to have slightly larger diameter than transseptal sheath outer rim. Although iatrogenic ASDs after TSP could frequently resolve spontaneously over time, large ASDs are sometimes unlikely to disappear thus interventional closure was required.^[8]^ A previous study demonstrated that 22% of patients treated with cryoballoon ablation with a 15-F transseptal sheath had persistent ASD after 11.6 months’ follow-up, which was even higher than those treated with double transseptal access using 2 8-F sheaths with radiofrequency catheter ablation.^[9]^ In present study, 18 patients underwent RF ablation used 8.5F SL1 sheath, and balloons with diameter of 4.0 mm were selected. The other 2 patients receiving cryoballoon ablation used 15-F sheath, and 5.0 mm diameter balloons were selected for ABS. Calculated perimeters for those balloons ranged from 12.6F to 15.7F, which were large enough to permit both sheaths to pass. Previous perspective research from Rillig studied 40 patients undergoing 8.5F SL-0 sheath or a 14F sheath for TSP and found that 79% of iASD closed spontaneously by 6 months.^[10]^ An even more optimal results from PROTECT-AF study showed that patients in whom a 12-F transseptal sheath was used to deploy LA appendage occlusion devices ASD remained in 11% at 6 months and 7% in a year.^[11,12]^ Another study detected a persistent ASD in one of the 2 patients with ABS had been performed through a GORE-TEX patch, and no evidence of residual ASD in all patients in which ABS had been performed^7^. In our study, no residual ASD was observed after 6 months of follow-up. These results indicated both efficacy and safety of this modified atrial balloon septoplasty method.

A more sophisticated procedure is radiofrequency needle which was invented directly to tackle difficult TSP.^[10]^ Procedural crossing success rate was higher with the RF needle approach as compared with the standard needle. Procedural times and degree of septum tenting favored the RF over standard needle.^[11]^ However, injury of septal wall imposed by Baylis RF energy needle might result in irreversible tissue damage and lifelong interatrial communication.^[13]^ Stiff headed guidewire PT Graphix (Boston Scientific) was used alone in other cases instead of the RF to penetrate interatrial septum by increased force, but there was a possibility in increased risk of penetrating walls of LA or PVs.^[2,18]^

TEE/intracardiac echocardiography was used in other studies to pinpoint the site of atrial septum and fossa ovalis.^[14,15]^ In our study, the procedure of balloon atrial septoplasty resembled coronary angioplasty. Interatrial septum was confirmed for ABS position by retrieving inflated balloon and injection of contrast under 2D fluoroscopy. BMW guidewire was commonly used in coronary intervention as its soft tip and elastinite body could guarantee no injury to the LA or PVs while providing enough support for TSP assembly to advance. The non-compliant small-caliber short balloon for coronary angioplasty (4.0 or 5.0 mm in diameter and 15 mm in length) used in our case was available in most catheterization rooms with coronary interventionists.

### 4.1 Study limitation

This was a retrospective study with a small sample size and a short mean follow-up duration, thus a large scale clinical trial is warranted for further application of this method. In addition, this modified ABS was performed only on patients with congenital septum, so it might not be generalized when a device is closed to interatrial septum and needles could not penetrate. This method was only use when conventional procedure of trans septal puncure failed, so there was no control group to analyze its proper efficacy. TEE/intracardiac echocardiography was not available in our catheterization room during the study. Lastly, location of trans septal apparatus was an approximation with 2D fluoroscopy which may lead to the septoplasty procedure performed at a relatively thick part of septum. The possibility of applying septoplasty in a thinner part of the septum was still unknown.

## 5 Conclusion

In AF patients with difficult LA septum, this modified percutaneous ABS could be a safe way to obtain transseptal access without short or long term complications.

## Author contributions

**Conceptualization:** Xiandong Yin.

**Data curation:** Lei Zhao, Ye Liu.

**Funding acquisition:** Xinchun Yang.

**Investigation:** Yuxing Wang.

**Project administration:** Xinchun Yang, Ye Liu.

**Resources:** Siyu Wang, Xinchun Yang.

**Supervision:** Xiandong Yin, Xinchun Yang.

**Writing – original draft:** Ye Liu.

**Writing – review & editing:** Siyu Wang, Lei Zhao.
